# Supplementary material for: Stimulation of endogenous cardioblasts by exogenous cell therapy after myocardial infarction
Source: EMBO Mol Med. 2014 May 5;6(6):760–77. doi: 10.1002/emmm.201303626 (PMC4203354; doi:10.1002/emmm.201303626)
Supplement: Supplementary file 1 — Supplementary Figure S1 [file emmm0006-0760-sd1.pdf]

Supp Fig 1

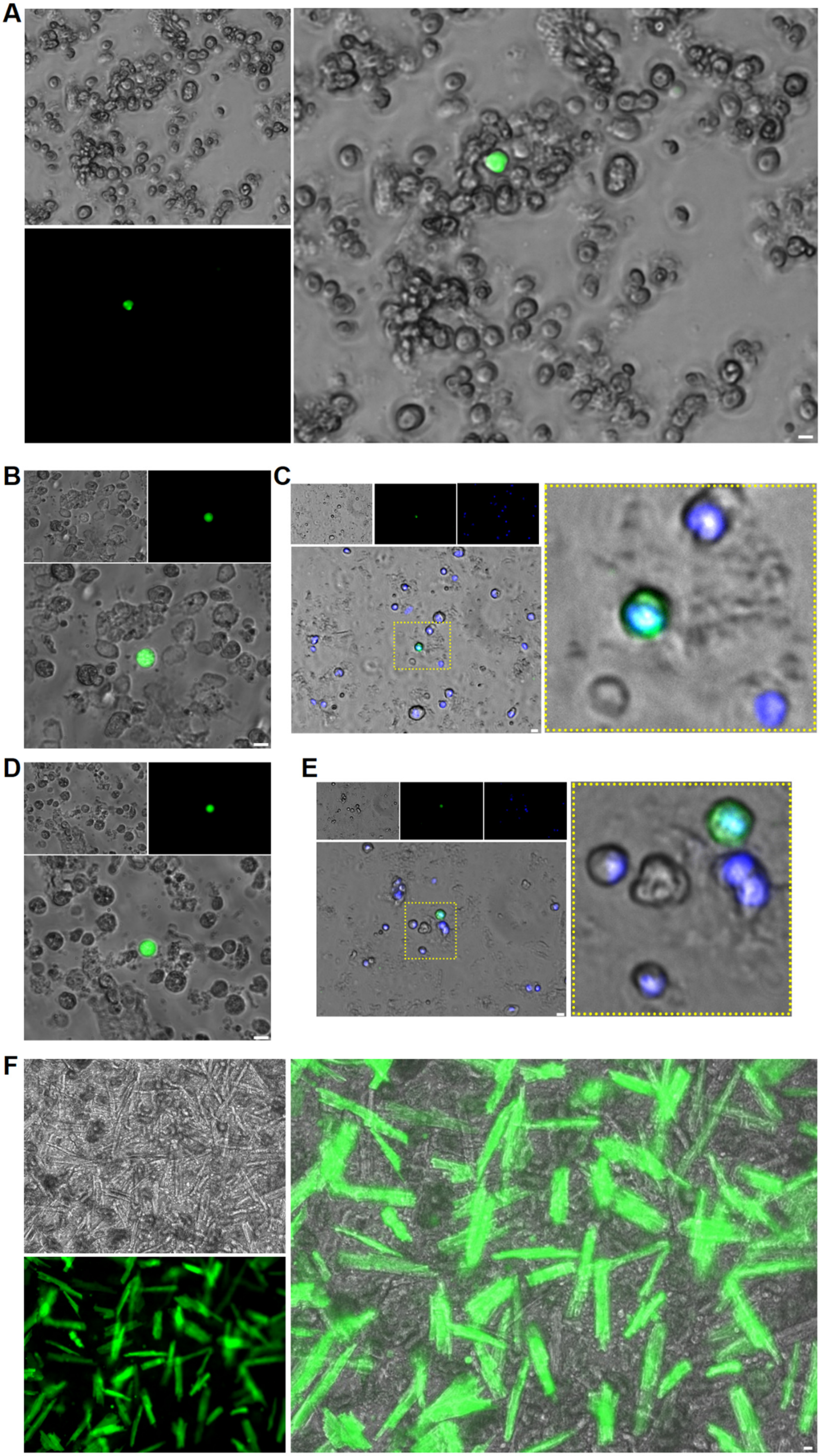

Supp Fig 1. Harsh enzymatic digestion of the explanted hearts followed by multiple filtering steps resulted in efficient myocyte depletion and isolation of non-cardiomyocyte cardiac cells. Note the absence of labeled cardiomyocytes in the myocyte-depleted cell preparations (**A-E**). Labeled cardiomyocytes were readily detectable after mild enzymatic dissociation of the heart on a Langendorff apparatus (**F**) (Blue: Hoechst, Green: GFP, scale bars: 10μm).
