# Supplementary material for: Stimulation of endogenous cardioblasts by exogenous cell therapy after myocardial infarction
Source: EMBO Mol Med. 2014 May 5;6(6):760–77. doi: 10.1002/emmm.201303626 (PMC4203354; doi:10.1002/emmm.201303626)
Supplement: Supplementary file 2 — Supplementary Figure S2 [file emmm0006-0760-sd2.pdf]

Supp Fig 2

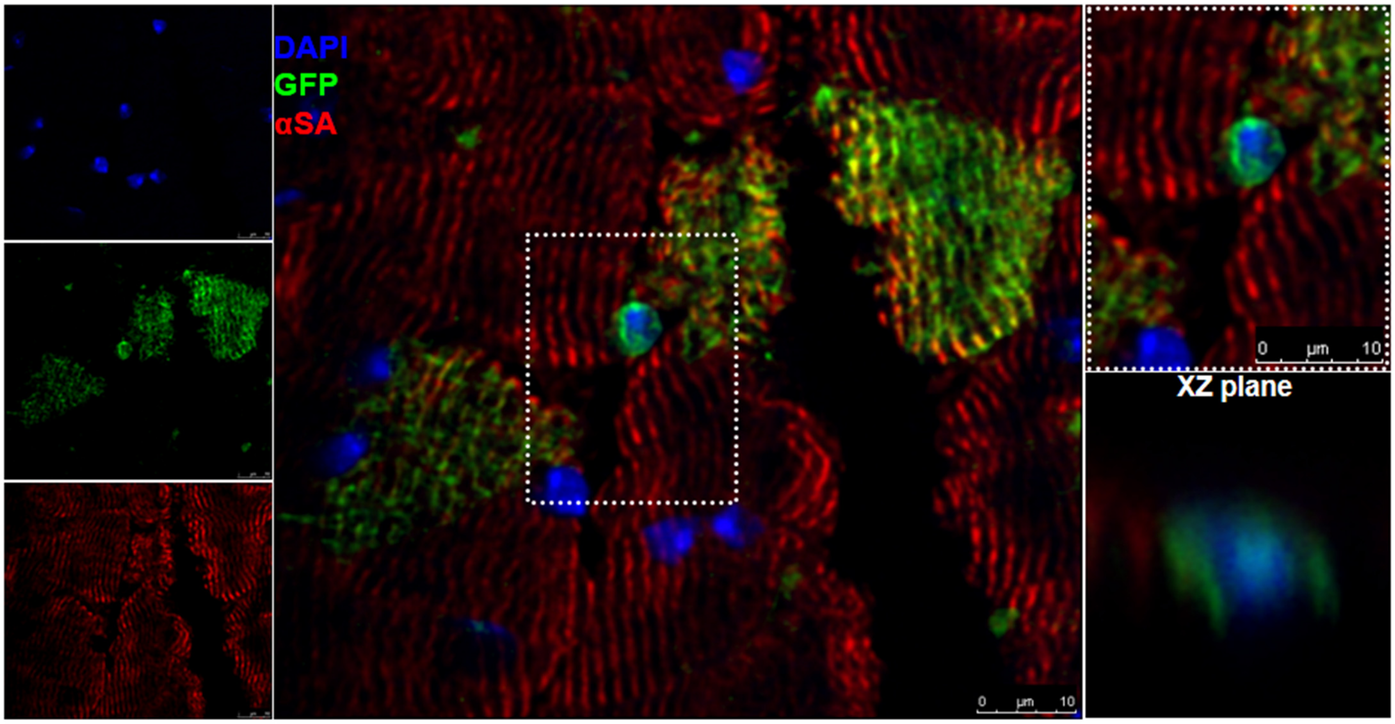

Supp Fig 2. Confocal microscopy in tissue sections from normal hearts revealed rare presence of GFP+ cardioblasts. Partial labeling of resident cardiomyocytes (which also express αMHC) is observed. Image on the right is a magnified image of boxed area on left. Image of confocal scanning across the XZ plane are also provided (blue: DAPI, green: GFP, red: αSA).
