# Supplementary material for: Stimulation of endogenous cardioblasts by exogenous cell therapy after myocardial infarction
Source: EMBO Mol Med. 2014 May 5;6(6):760–77. doi: 10.1002/emmm.201303626 (PMC4203354; doi:10.1002/emmm.201303626)
Supplement: Supplementary file 3 — Supplementary Figure S3 [file emmm0006-0760-sd3.pdf]

Supp Fig 3

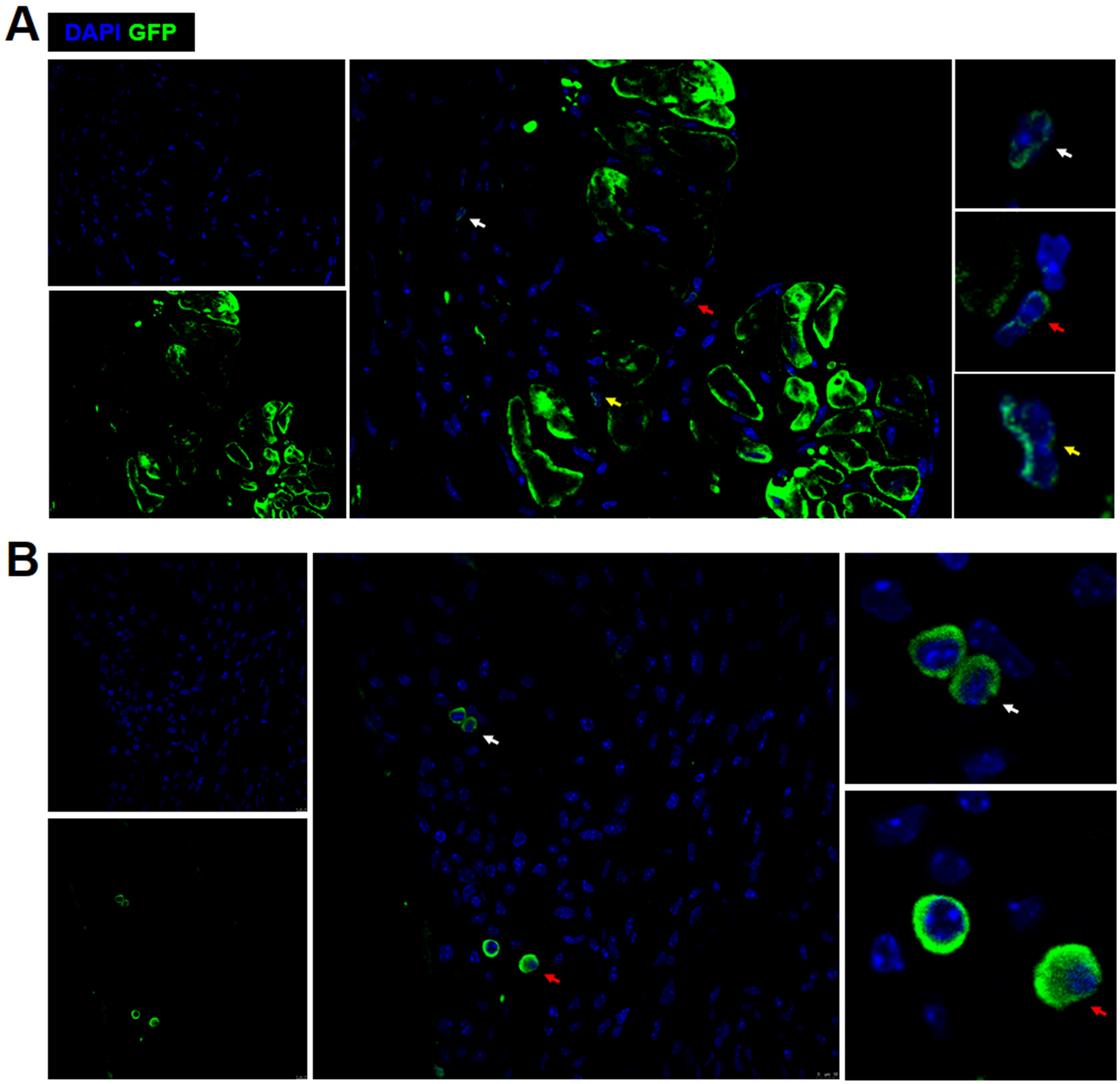

Supp Fig 3. Confocal microscopy in tissue sections from infarcted hearts (without [A] and post-CDC therapy [B] revealed increased presence of GFP+ cardioblasts in the infarct area. Partial labeling of resident cardiomyocytes (which also express αMHC) is observed. The area of the infarct is identified by the lack of cardiomyocytes (negative for GFP) and the presence of small non-myocyte GFP-/DAPI+ cells. Images on the right are magnified images of GFP+ cardioblasts marked by arrows on left (blue: DAPI, green: GFP).
