# Supplementary material for: Stimulation of endogenous cardioblasts by exogenous cell therapy after myocardial infarction
Source: EMBO Mol Med. 2014 May 5;6(6):760–77. doi: 10.1002/emmm.201303626 (PMC4203354; doi:10.1002/emmm.201303626)
Supplement: Supplementary file 4 — Supplementary Figure S4 [file emmm0006-0760-sd4.pdf]

Supp Fig 4

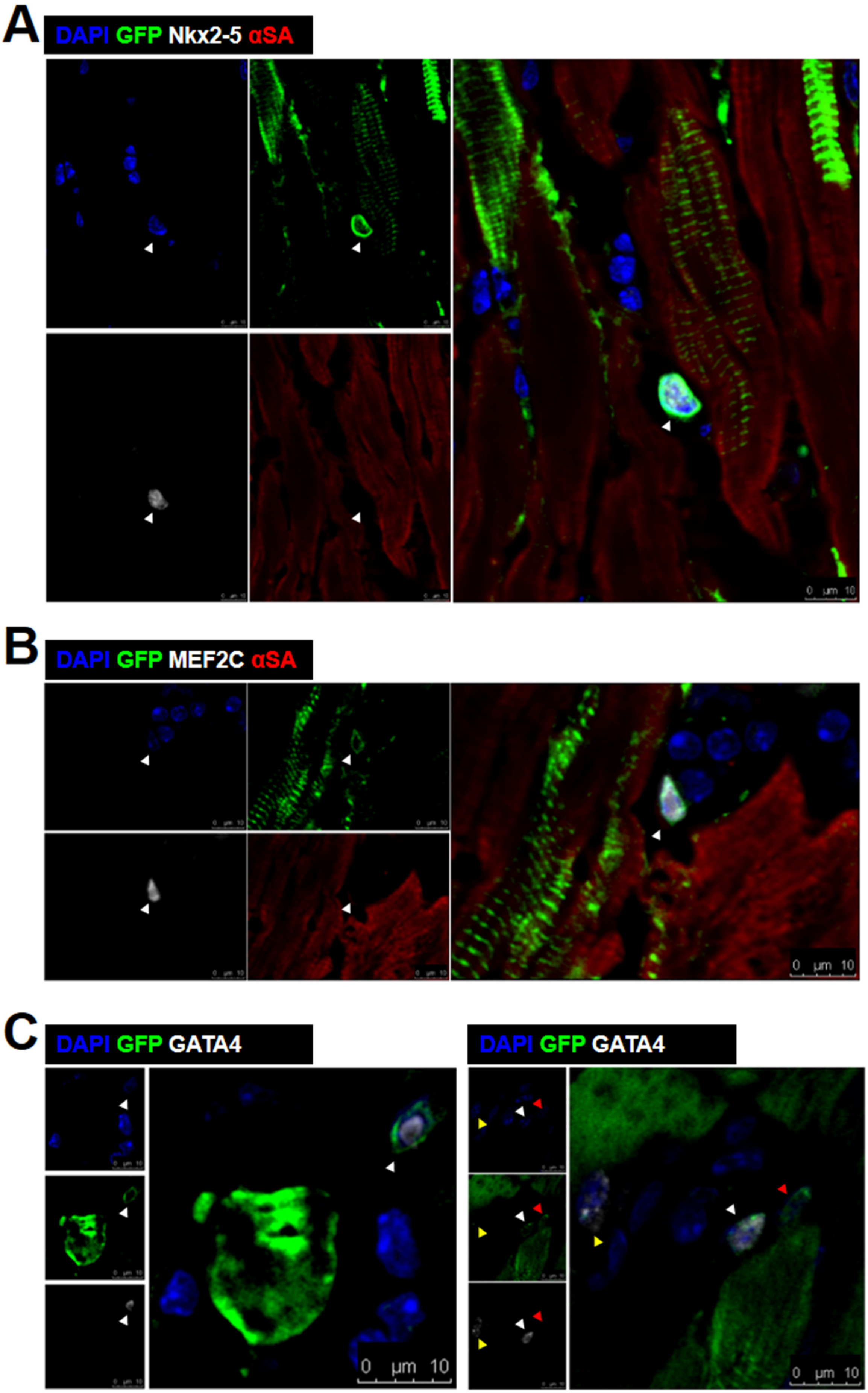
A
DAPI GFP Nkx2-5 αSA
0 µm 10
B
DAPI GFP MEF2C αSA
0 µm 10
C
DAPI GFP GATA4
0 µm 10
DAPI GFP GATA4
0 µm 10

Supp Fig 4. Fluorescent tissue immunohistochemistry reveals GFP+, αSA- cardioblasts expressing NKX2-5 (A), MEF2C (B) and GATA4 (C) (white arrows). Red arrow in C denotes a GFP+/GATA4- cardioblast. Yellow arrow in C denotes a GFP-/GATA4+ cell (blue: DAPI, green: GFP, red: αSA, white: NKX2-5/MEF2C/GATA4).
