# Supplementary material for: Stimulation of endogenous cardioblasts by exogenous cell therapy after myocardial infarction
Source: EMBO Mol Med. 2014 May 5;6(6):760–77. doi: 10.1002/emmm.201303626 (PMC4203354; doi:10.1002/emmm.201303626)
Supplement: Supplementary file 5 — Supplementary Figure S5 [file emmm0006-0760-sd5.pdf]

Supp Fig 5

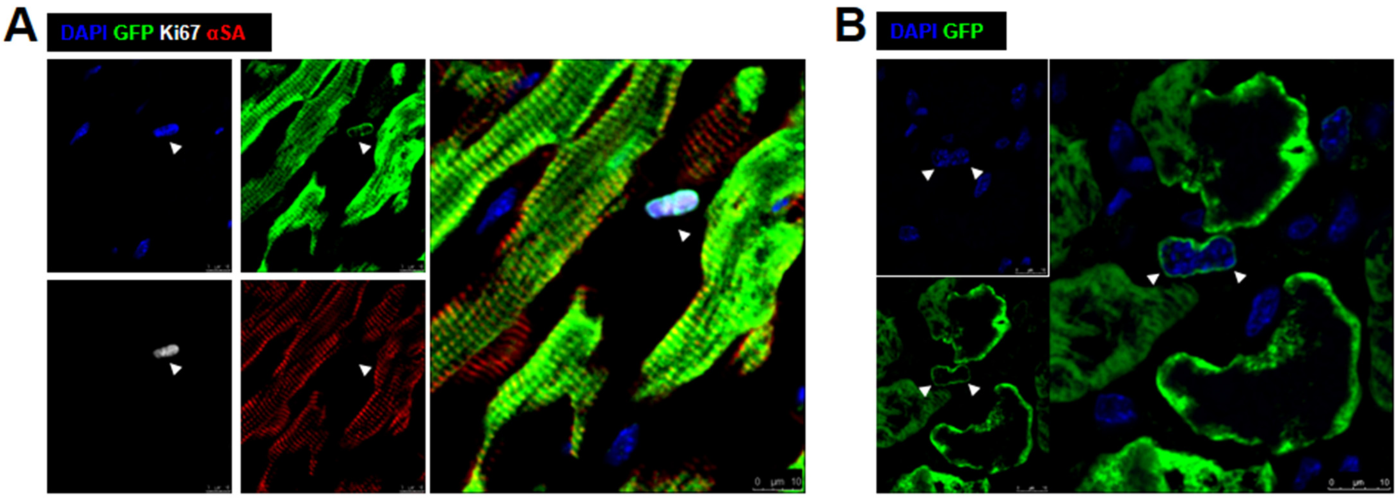

Supp Fig 5. A: Confocal microscopy of tissue sections from infarcted hearts revealed presence of Ki67+/GFP+ cardioblasts in the border zone (arrow). B: Confocal microscopy of tissue sections from infarcted hearts revealed presence of GFP+ cardioblasts appearing to undergo mitosis in the border zone (arrows) (blue: DAPI, green: GFP, red: αSA, white: Ki67).
