# Supplementary figures and images for: Stimulation of endogenous cardioblasts by exogenous cell therapy after myocardial infarction
Source: EMBO Mol Med. 2014 May 5;6(6):760–77. doi: 10.1002/emmm.201303626 (PMC4203354; doi:10.1002/emmm.201303626)

**Supp Fig 6**

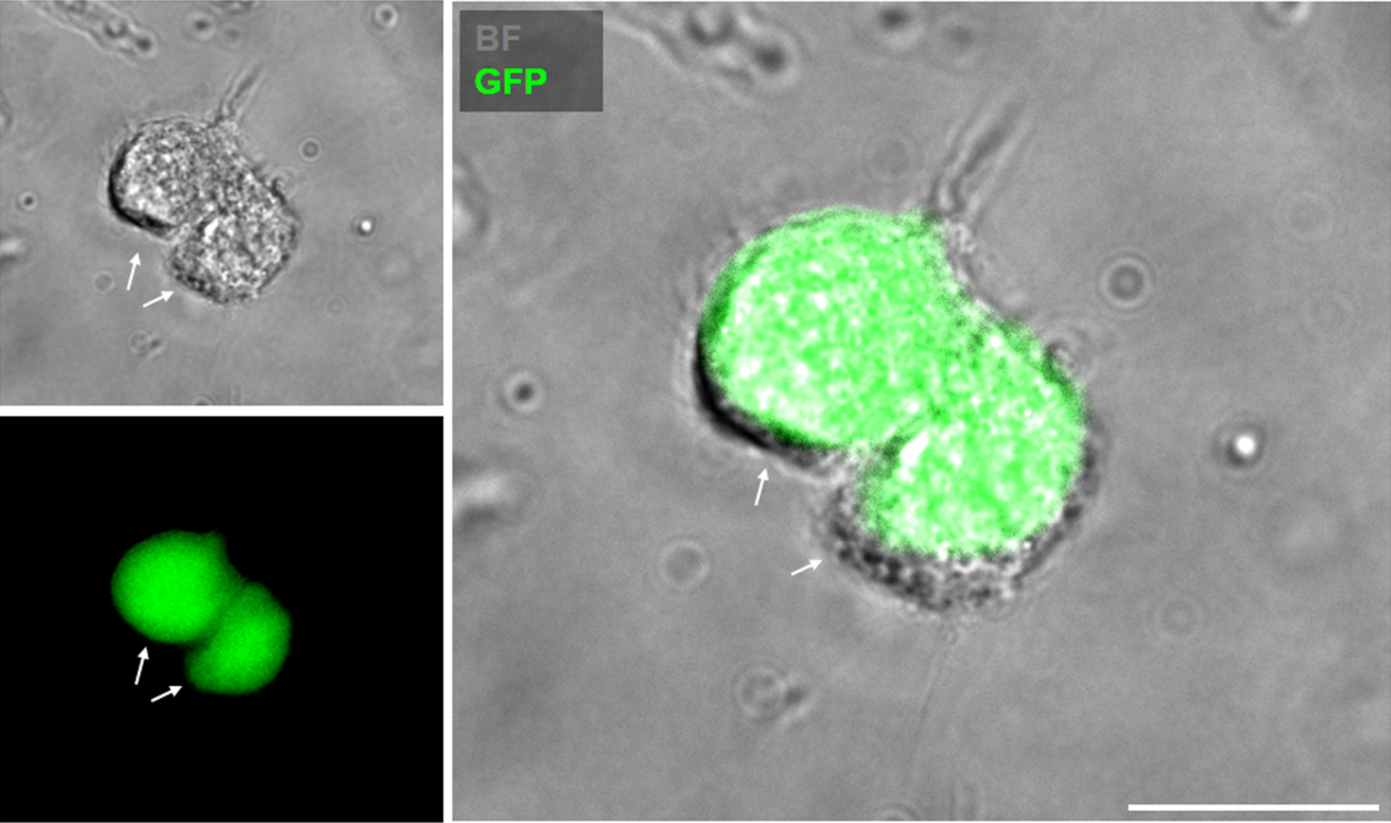

**Supp Fig 6.** A rare mitotic GFP+ CPC in culture (bright field (BF), green: GFP) (scale bar: 10μm).

Supplement: Supplementary file 6 — Supplementary Figure S6 [file emmm0006-0760-sd6.pdf]
