# Supplementary material for: Stimulation of endogenous cardioblasts by exogenous cell therapy after myocardial infarction
Source: EMBO Mol Med. 2014 May 5;6(6):760–77. doi: 10.1002/emmm.201303626 (PMC4203354; doi:10.1002/emmm.201303626)
Supplement: Supplementary file 7 — Supplementary Figure S7 [file emmm0006-0760-sd7.pdf]

Supp Fig 7

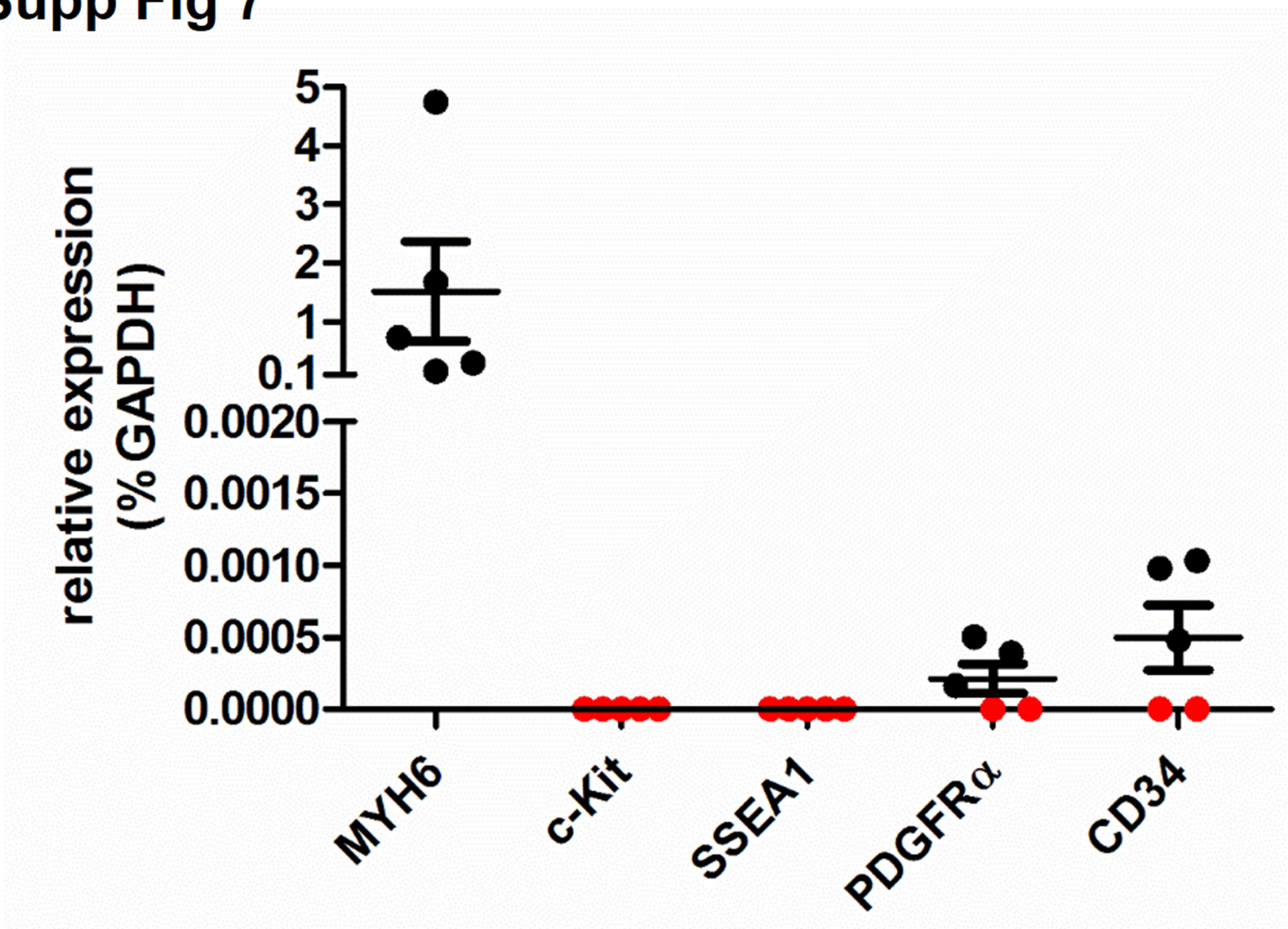

Supp Fig 7. Gene expression of KIT (c-Kit), FUT4 (SSEA1), PDGFRa and CD34 in FACS-sorted GFP+ myocyte-depleted cardiac cells (obtained from 5 hearts). Expression of KIT (c-Kit) and FUT4 (SSEA1) was not detected in any samples. Very low expression of PDGRFα and CD34 was detected in 3/5 samples (on average >1000 fold less compared to expression of MYH6 [αMHC] in the same samples). Expression is each gene is presented as % expression of GAPDH. Red dots indicate undetected expression.
