# Supplementary material for: Stimulation of endogenous cardioblasts by exogenous cell therapy after myocardial infarction
Source: EMBO Mol Med. 2014 May 5;6(6):760–77. doi: 10.1002/emmm.201303626 (PMC4203354; doi:10.1002/emmm.201303626)
Supplement: Supplementary file 8 — Supplementary Figure S8 [file emmm0006-0760-sd8.pdf]

Supp Fig 8

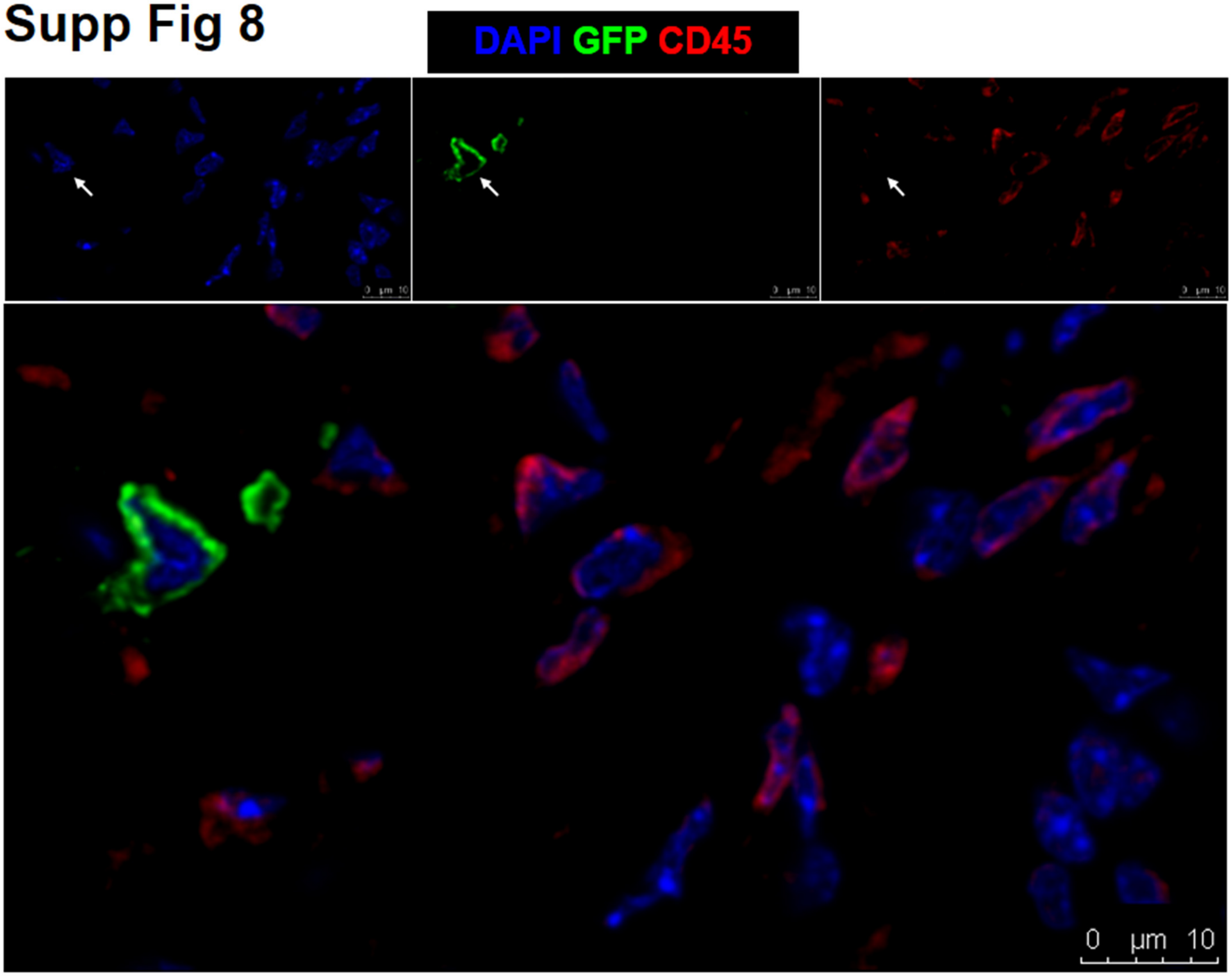

Supp Fig 8. Confocal microscopy in tissue sections from infarcted hearts revealed that GFP+ cardioblasts were CD45 negative (blue: DAPI, green: GFP, red: CD45).
