# Supplementary material for: Stimulation of endogenous cardioblasts by exogenous cell therapy after myocardial infarction
Source: EMBO Mol Med. 2014 May 5;6(6):760–77. doi: 10.1002/emmm.201303626 (PMC4203354; doi:10.1002/emmm.201303626)
Supplement: Supplementary file 9 — Supplementary Figure S9 [file emmm0006-0760-sd9.pdf]

Supp Fig 9

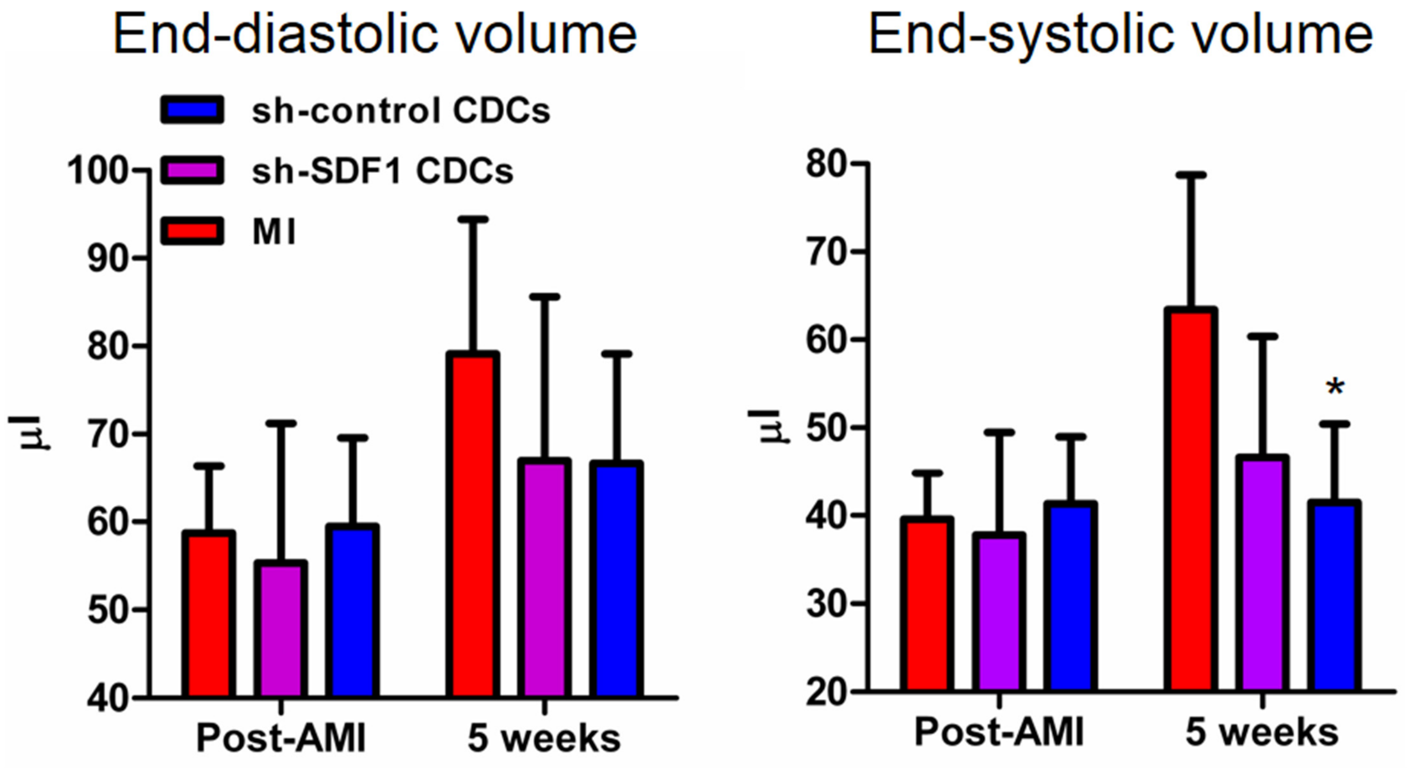

Supp Fig 9. Echocardiographic assessment of end-diastolic and end-systolic volumes (n=5 mice/group).
