# Supplementary material for: Stimulation of endogenous cardioblasts by exogenous cell therapy after myocardial infarction
Source: EMBO Mol Med. 2014 May 5;6(6):760–77. doi: 10.1002/emmm.201303626 (PMC4203354; doi:10.1002/emmm.201303626)
Supplement: Supplementary file 10 — Supplementary Figure S10 [file emmm0006-0760-sd10.pdf]

Supp Fig 10

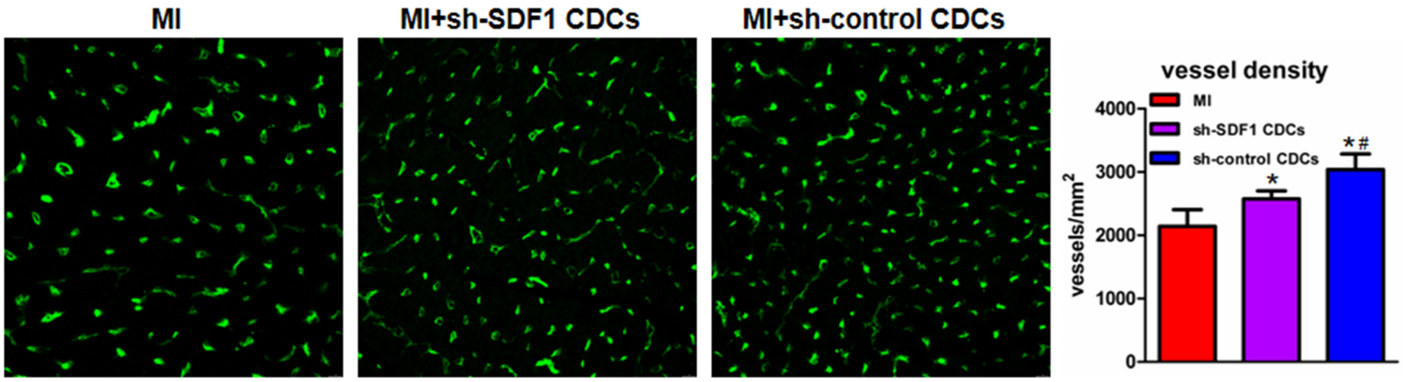

Supp Fig 10. Vessel density, as assessed by isolectin staining (green) in areas of viable myocardium in the infarct border zone, was decreased after knock-down of SDF1 in transplanted CDCs (n=4-5 mice/group).
