# Supplementary material for: Stimulation of endogenous cardioblasts by exogenous cell therapy after myocardial infarction
Source: EMBO Mol Med. 2014 May 5;6(6):760–77. doi: 10.1002/emmm.201303626 (PMC4203354; doi:10.1002/emmm.201303626)
Supplement: Supplementary file 11 — Supplementary Table S1 [file emmm0006-0760-sd11.pdf]

Experiment	Statistical test	P value
Fig 1C (FC)	Independent samples t-test	0.001
Fig 1C (epi)	Independent samples t-test	0.030
Fig 1H	Independent samples t-test	<0.001
Fig 3 (NKX2-5)		
GFP- vs GFP+	Mann-Whitney U test	0.008
GFP+ vs myocytes	Mann-Whitney U test	0.036
myocytes vs GFP-	Mann-Whitney U test	0.036
Fig 3 (MEF2C)		
GFP- vs GFP+	Mann-Whitney U test	0.008
GFP+ vs myocytes	Mann-Whitney U test	0.786
myocytes vs GFP-	Mann-Whitney U test	0.036
Fig 3 (TBX3)		
GFP- vs GFP+	Mann-Whitney U test	0.008
GFP+ vs myocytes	Mann-Whitney U test	0.143
myocytes vs GFP-	Mann-Whitney U test	0.036
Fig 3 (GATA4)		
GFP- vs GFP+	Mann-Whitney U test	0.151
GFP+ vs myocytes	Mann-Whitney U test	0.786
myocytes vs GFP-	Mann-Whitney U test	0.571
Fig 3 (BMP2)		
GFP- vs GFP+	Mann-Whitney U test	0.008
GFP+ vs myocytes	Mann-Whitney U test	0.036
myocytes vs GFP-	Mann-Whitney U test	0.036

Fig 3 (FOXC2)		
GFP- vs GFP+	Mann-Whitney U test	0.008
GFP+ vs myocytes	Mann-Whitney U test	0.250
myocytes vs GFP-	Mann-Whitney U test	0.571
Fig 3 (MYH6)		
GFP- vs GFP+	Mann-Whitney U test	0.008
GFP+ vs myocytes	Mann-Whitney U test	0.036
myocytes vs GFP-	Mann-Whitney U test	0.036
Fig 3 (MYH7)		
GFP- vs GFP+	Mann-Whitney U test	0.008
GFP+ vs myocytes	Mann-Whitney U test	0.571
myocytes vs GFP-	Mann-Whitney U test	0.036
Fig 3 (MYL2)		
GFP- vs GFP+	Mann-Whitney U test	0.008
GFP+ vs myocytes	Mann-Whitney U test	0.036
myocytes vs GFP-	Mann-Whitney U test	0.036
Fig 3 (MYL3)		
GFP- vs GFP+	Mann-Whitney U test	0.008
GFP+ vs myocytes	Mann-Whitney U test	0.036
myocytes vs GFP-	Mann-Whitney U test	0.036
Fig 3 (TNNI3)		
GFP- vs GFP+	Mann-Whitney U test	0.008
GFP+ vs myocytes	Mann-Whitney U test	0.036
myocytes vs GFP-	Mann-Whitney U test	0.036

Fig 3 (TNNT2)		
GFP- vs GFP+	Mann-Whitney U test	0.008
GFP+ vs myocytes	Mann-Whitney U test	0.071
myocytes vs GFP-	Mann-Whitney U test	0.036
Fig 3 (MYBPC3)		
GFP- vs GFP+	Mann-Whitney U test	0.008
GFP+ vs myocytes	Mann-Whitney U test	0.036
myocytes vs GFP-	Mann-Whitney U test	0.036
Fig 3 (PLN)		
GFP- vs GFP+	Mann-Whitney U test	0.008
GFP+ vs myocytes	Mann-Whitney U test	0.036
myocytes vs GFP-	Mann-Whitney U test	0.036
Fig 3 (DES)		
GFP- vs GFP+	Mann-Whitney U test	0.008
GFP+ vs myocytes	Mann-Whitney U test	0.036
myocytes vs GFP-	Mann-Whitney U test	0.036
Fig 3 (NPPA)		
GFP- vs GFP+	Mann-Whitney U test	0.008
GFP+ vs myocytes	Mann-Whitney U test	0.036
myocytes vs GFP-	Mann-Whitney U test	0.036
Fig 3 (CKM)		
GFP- vs GFP+	Mann-Whitney U test	0.008
GFP+ vs myocytes	Mann-Whitney U test	0.036
myocytes vs GFP-	Mann-Whitney U test	0.036

Fig 3 (TTN)<br><br>GFP- vs GFP+<br><br>GFP+ vs myocytes<br><br>myocytes vs GFP-	 <br>Mann-Whitney U test<br><br>Mann-Whitney U test<br><br>Mann-Whitney U test	 <br>0.008<br><br>0.036<br><br>0.036
Fig 3 (VIM)<br><br>GFP- vs GFP+<br><br>GFP+ vs myocytes<br><br>myocytes vs GFP-	 <br>Mann-Whitney U test<br><br>Mann-Whitney U test<br><br>Mann-Whitney U test	 <br>0.008<br><br>0.250<br><br>0.036
Fig 3 (VWF)<br><br>GFP- vs GFP+<br><br>GFP+ vs myocytes<br><br>myocytes vs GFP-	 <br>Mann-Whitney U test<br><br>Mann-Whitney U test<br><br>Mann-Whitney U test	 <br>0.008<br><br>0.571<br><br>0.036
Fig 3 (ACTA2)<br><br>GFP- vs GFP+<br><br>GFP+ vs myocytes<br><br>myocytes vs GFP-	 <br>Mann-Whitney U test<br><br>Mann-Whitney U test<br><br>Mann-Whitney U test	 <br>0.036<br><br>0.400<br><br>0.036
Fig 3 (ISL1) (not shown in Fig 3)<br><br>GFP- vs GFP+	Mann-Whitney U test	0.151
Fig 3 (TBX2) (not shown in Fig)<br><br>GFP- vs GFP+	Mann-Whitney U test	0.690
Fig 3 (TBX5) (not shown in Fig)<br><br>GFP- vs GFP+	Mann-Whitney U test	0.690
Fig 3 (TBX20) (not shown in Fig)	Mann-Whitney U test	0.690

GFP- vs GFP+		
Fig 3 (MESP1) (not shown in Fig)<br><br>GFP- vs GFP+	Mann-Whitney U test	0.690
Fig 3 (MESP2) (not shown in Fig)<br><br>GFP- vs GFP+	Mann-Whitney U test	0.690
Fig 3 (GATA6) (not shown in Fig)<br><br>GFP- vs GFP+	Mann-Whitney U test	0.151
Fig 3 (WNT2) (not shown in Fig)<br><br>GFP- vs GFP+	Mann-Whitney U test	0.690
Fig 3 (WNT5a) (not shown in Fig)<br><br>GFP- vs GFP+	Mann-Whitney U test	0.690
Fig 3 (WNT11) (not shown in Fig)<br><br>GFP- vs GFP+	Mann-Whitney U test	0.690
Fig 3 (DKK1) (not shown in Fig)<br><br>GFP- vs GFP+	Mann-Whitney U test	0.690
Fig 3 (SMARCD3) (not shown in Fig)<br><br>GFP- vs GFP+	Mann-Whitney U test	0.151
Fig 3 (PDGFRA) (not shown in Fig)<br><br>GFP- vs GFP+	Mann-Whitney U test	0.690
Fig 3 (FOXC1) (not shown in Fig)<br><br>GFP- vs GFP+	Mann-Whitney U test	0.690
Fig 3 (HAND1) (not shown in Fig)<br><br>GFP- vs GFP+	Mann-Whitney U test	0.690
Fig 3 (HAND2) (not shown in Fig)	Mann-Whitney U test	0.151

GFP- vs GFP+		
Fig 6C (FC)	Independent samples t-test	0.895
Fig 6C (epi)	Independent samples t-test	n/a (SDs=0)
Fig 6F (FC)	Independent samples t-test	0.006
Fig 6F (epi)	Independent samples t-test	0.014
Fig 7A (FC) sham vs MI	ANOVA (LSD)	<0.001 (0.022)
Fig 7A (FC) sham vs CDCs	ANOVA (LSD)	<0.001 (<0.001)
Fig 7A (FC) MI vs CDCs	ANOVA (LSD)	<0.001 (<0.001)
Fig 7A (epi) sham vs MI	ANOVA (LSD)	0.001 (0.237)
Fig 7A (epi) sham vs CDCs	ANOVA (LSD)	0.001 (0.001)
Fig 7A (epi) MI vs CDCs	ANOVA (LSD)	0.001 (0.001)
Fig 7D	Independent samples t-test	<0.001
Fig 7E	Independent samples t-test	<0.001
Fig 7F MI vs sh-control	ANOVA (LSD)	0.048 (0.023)
Fig 7F MI vs sh-SDF1	ANOVA (LSD)	0.048 (0.620)
Fig 7F sh-SDF1 vs sh-control	ANOVA (LSD)	0.048 (0.046)
Fig 7F MI vs sh-control	ANOVA (LSD)	0.016 (0.006)
Fig 7F MI vs sh-VEGF	ANOVA (LSD)	0.016 (0.229)
Fig 7F sh-VEGF vs sh-control	ANOVA (LSD)	0.016 (0.032)
Fig 7H (FC) sh-control vs sh-SDF1	ANOVA (LSD)	0.020 (0.008)
Fig 7H (FC) sh-control vs sh-VEGF	ANOVA (LSD)	0.020 (0.441)
Fig 7H (FC) sh-SDF1 vs sh-VEGF	ANOVA (LSD)	0.020 (0.034)
Fig 7H (epi) sh-control vs sh-SDF1	ANOVA (LSD)	0.035 (0.015)
Fig 7H (epi) sh-control vs sh-VEGF	ANOVA (LSD)	0.035 (0.577)

Fig 7H (epi) sh-SDF1 vs sh-VEGF	ANOVA (LSD)	0.035 (0.043)
Fig 8B MI vs sh-SDF1	ANOVA (LSD)	0.003 (0.001)
Fig 8B MI vs sh-control	ANOVA (LSD)	0.003 (0.005)
Fig 8B sh-SDF1 vs sh-control	ANOVA (LSD)	0.003 (0.494)
Fig 8C MI vs sh-SDF1	ANOVA (LSD)	<0.001 (<0001)
Fig 8C MI vs sh-control	ANOVA (LSD)	<0.001(<0001)
Fig 8C sh-SDF1 vs sh-control	ANOVA (LSD)	<0.001 (0.133)
Fig 8D MI vs sh-SDF1	ANOVA (LSD)	0.001 (0.010)
Fig 8D MI vs sh-control	ANOVA (LSD)	0.001 (<0.001)
Fig 8D sh-SDF1 vs sh-control	ANOVA (LSD)	0.001 (0.048)
Fig 8E MI vs sh-SDF1	ANOVA (LSD)	0.002 (0.033)
Fig 8E MI vs sh-control	ANOVA (LSD)	0.002 (<0.001)
Fig 8E sh-SDF1 vs sh-control	ANOVA (LSD)	0.002 (0.031)
Fig 8F (post-AMI) MI vs sh-SDF1	ANOVA (LSD)	0.741
Fig 8F (post-AMI) MI vs sh-control	ANOVA (LSD)	0.741
Fig 8F (post-AMI) sh-SDF1 vs sh-control	ANOVA (LSD)	0.741
Fig 8F (5w) MI vs sh-SDF1	ANOVA (LSD)	<0.001 (0.005)
Fig 8F (5w) MI vs sh-control	ANOVA (LSD)	<0.001 (<0.001)
Fig 8F (5w) sh-SDF1 vs sh-control	ANOVA (LSD)	<0.001 (0.031)
Fig 8G sham vs MI	ANOVA (LSD)	<0.001 (0.002)
Fig 8G sham vs sh-SDF1	ANOVA (LSD)	<0.001 (<0.001)
Fig 8G sham vs sh-control	ANOVA (LSD)	<0.001 (<0.001)
Fig 8G MI vs sh-SDF1	ANOVA (LSD)	<0.001 (0.024)
Fig 8G MI vs sh-control	ANOVA (LSD)	<0.001 (<0.001)

Fig 8G sh-SDF1 vs sh-control	ANOVA (LSD)	<0.001 (0.008)
Supp Fig 9 EDV (post-AMI) MI vs sh-SDF1	ANOVA (LSD)	0.838
Supp Fig 9 EDV (post-AMI) MI vs sh-control	ANOVA (LSD)	0.838
Supp Fig 9 EDV (post-AMI) sh-SDF1 vs sh-control	ANOVA (LSD)	0.838
Supp Fig 9 EDV (5w) MI vs sh-SDF1	ANOVA (LSD)	0.815
Supp Fig 9 EDV (5w) MI vs sh-control	ANOVA (LSD)	0.815
Supp Fig 9 EDV (5w) sh-SDF1 vs sh-control	ANOVA (LSD)	0.815
Supp Fig 9 ESV (post-AMI) MI vs sh-SDF1	ANOVA (LSD)	0.386
Supp Fig 9 ESV (post-AMI) MI vs sh-control	ANOVA (LSD)	0.386
Supp Fig 9 ESV (post-AMI) sh-SDF1 vs sh-control	ANOVA (LSD)	0.386
Supp Fig 9 ESV (5w) MI vs sh-SDF1	ANOVA (LSD)	0.048 (0.062)
Supp Fig 9 ESV (5w) MI vs sh-control	ANOVA (LSD)	0.048 (0.020)
Supp Fig 9 ESV (5w) sh-SDF1 vs sh-control	ANOVA (LSD)	0.048 (0.541)
Supp Fig 10 MI vs sh-SDF1	ANOVA (LSD)	<0.001 (0.021)
Supp Fig 10 MI vs sh-control	ANOVA (LSD)	<0.001 (<0.001)
Supp Fig 10 sh-SDF1 vs sh-control	ANOVA (LSD)	<0.001 (0.011)

Table S1. Exact p values and the specific statistical test performed for each experiment.
