# Supplementary material for: Stimulation of endogenous cardioblasts by exogenous cell therapy after myocardial infarction
Source: EMBO Mol Med. 2014 May 5;6(6):760–77. doi: 10.1002/emmm.201303626 (PMC4203354; doi:10.1002/emmm.201303626)
Supplement: Supplementary file 16 — Supplementary Movie Legends [file emmm0006-0760-sd16.pdf]

Movie S1. Video of a CPC exhibiting spontaneous contractile activity *in vitro* after 1 day in culture. The corresponding still images (bright field, GFP and merged image) are provided in Fig 2A (left).

Movie S2. Video of a CPC exhibiting spontaneous contractile activity *in vitro* after 1 day in culture. The corresponding still images (bright field, GFP and merged image) are provided in Fig 2A (right).
